# Drone-Based Environmental Monitoring and Image Processing Approaches for Resource Estimates of Private Native Forest

**DOI:** 10.3390/s22207872

**Published:** 2022-10-17

**Authors:** Sanjeev Kumar Srivastava, Kah Phooi Seng, Li Minn Ang, Anibal ‘Nahuel’ A. Pachas, Tom Lewis

**Affiliations:** 1 School of Science Technology and Engineering, University of the Sunshine Coast, Sippy Downs, QLD 4556, Australia; 2 School of AI and Advanced Computing, Xi’an Jiaotong Liverpool University, Suzhou 215000, China; 3 School of Computer Science, Queensland University of Technology, Brisbane City, QLD 4000, Australia; 4 Department of Agriculture and Fisheries, Queensland Government, 1 Cartwright Road, Gympie, QLD 4570, Australia

**Keywords:** digital photogrammetry, drone-based monitoring, forest resource estimation, image analysis, private native forests, remote sensing

## Abstract

This paper investigated the utility of drone-based environmental monitoring to assist with forest inventory in Queensland private native forests (PNF). The research aimed to build capabilities to carry out forest inventory more efficiently without the need to rely on laborious field assessments. The use of drone-derived images and the subsequent application of digital photogrammetry to obtain information about PNFs are underinvestigated in southeast Queensland vegetation types. In this study, we used image processing to separate individual trees and digital photogrammetry to derive a canopy height model (CHM). The study was supported with tree height data collected in the field for one site. The paper addressed the research question “How well do drone-derived point clouds estimate the height of trees in PNF ecosystems?” The study indicated that a drone with a basic RGB camera can estimate tree height with good confidence. The results can potentially be applied across multiple land tenures and similar forest types. This informs the development of drone-based and remote-sensing image-processing methods, which will lead to improved forest inventories, thereby providing forest managers with recent, accurate, and efficient information on forest resources.

## 1. Introduction

There are approximately 2 million hectares of potentially harvestable private native forest (PNF) in southern Queensland. However, there is little information on the productive state (in terms of timber production) of this resource. Field surveys suggest that large parts of this resource are in a relatively unproductive condition. However, some works have shown that management (e.g., tree thinning) can greatly enhance the productive value of the native forests and that remote-sensing options can provide useful information on the heights and species within native forest environments. There is a need for forest inventory work to determine where forest management might be best targeted. If well managed, this resource has great potential for helping maintain or grow the hardwood timber industry in Queensland. Image processing and remote sensing have the potential to increase cost-effectiveness and time efficiency and reduce uncertainty compared with the traditional method of generating forest inventory data from sampling plots [1,2,3]. Accurate, spatially detailed forestry inventories can be generated from cost-effective, near real-time data sets [4,5,6].

In the literature, four remote sensing approaches have generally been used to monitor forest environments: (1) airborne and terrestrial light detection and ranging (LiDAR); (2) interferometric radar; (3) high-resolution multispectral images, and (4) photogrammetry [7,8,9]. LiDAR (light detection and ranging) data and high-resolution remote-sensing images (e.g., photogrammetry) can be used to develop a canopy point cloud. Where the density of this point cloud is high enough (i.e., high spatial resolution), this allows for the determination of the number of trees per hectare and their canopy heights. However, flying over potential forests of interest to obtain LiDAR data is cost-prohibitive, and while high-resolution satellite images are useful for giving an indication of the overall canopy height, this method of photogrammetry does not allow the determination of the number of trees per hectare (point clouds derived are not of sufficient density) in the forest. Motion photogrammetry using unmanned aerial vehicles (i.e., drones) can produce high-density point clouds to allow the estimation of productive tree crowns. However, to the best of our knowledge, this method has not been validated in subtropical eucalypt forests.

Forest inventory data collection is enhanced by recent developments in UAV image collection, and products using digital photogrammetry are cost-effective options for forest structure profiling [10]). There are several examples of UAV-supported remote-sensing techniques that have been used to study forests [11,12]. Several studies have demonstrated the accuracy of aerial laser survey (ALS) or LiDAR for providing forest inventory data [13,14,15,16,17] and its efficiency compared with field measurements [18]. Yet ALS can be cost-prohibitive, especially when analysing temporal change. The high cost associated with ALS can limit the spatial extent and temporal frequency of data collection [19,20,21]. Drone-based approaches have several advantages for forest monitoring. Lower flight altitudes are able to give high spatial–temporal resolution, data acquisition frequency can be high due to the relatively low operating costs, and the algorithms associated with UAV-derived products are robust and provide accurate elevation data. 

This paper investigates the utility of drone-based monitoring and image processing approaches to assist with forest inventory in Queensland native forests. The research aims to build capabilities to carry out forest inventory far more efficiently without the need to rely on laborious field assessments. In this paper, we used a number of existing private native forest sites where field inventory data was available. We investigated the utility of drone-based remote-sensing and image-processing data for the collection of forest inventory information. The findings of this study will be highly beneficial for various agencies working on managing natural and commercial forests. This paper is structured as follows. Section 2 discusses some previous works on drone-based approaches for environmental and forest monitoring. Section 3 describes the methodology and data collection approaches used for the investigation. Section 4 discusses the results and the interpretation of the results. Some concluding remarks are given in Section 5.

## 2. Recent Works on Drone-Based Approaches for Environmental Monitoring

The objective of this section is to provide background information before we discuss our proposed approach for drone-based environmental monitoring and image processing to assist with forest inventory applied to the private native forests in southern Queensland. Table 1 shows a summary of the works. We give some examples for two categories that commonly use drone-based environmental monitoring: (1) land monitoring and identification and (2) aquatic/marine monitoring and identification. The work proposed in this paper is for land-based monitoring and identification, with a focus on utilizing drone-based imagery for forest inventory.

## 3. Methodology and Data Collection

### 3.1. Site Selection for Drone Monitoring

This research used two privately owned sites where the native forest is managed for timber and grazing production. The drone data were collected for different sites in the period from August to November 2020 and the field–forest inventory data were collected at the same time. The research utilised point clouds derived from drone flights over the selected sites. The following software packages were used for extracting information: (1) ArcGIS Pro/ArcGIS; (2) R-statistics; (3) ENVI; and (4) Metashape.

Images were collected using a Phantom 4 pro drone with 4Kcamera in red, green and blue spectrum during August-September 2020. The drone was flown in a Double-Grid PIX4D Capture pattern, at an altitude of 60 m above ground level (AGL), with a 65° camera angle and 80% lateral and longitudinal image overlap. Flights were carried out in accordance with Australia’s Civil Aviation Safety Authority (CASA) regulations for safe operation of unmanned aerial vehicles (UAVs) and by suitably qualified personnel in clear weather conditions with maximum winds not exceeding 20 kph. Images were stored on a 32 GB micro SD card mounted on the drone and subsequently used for analysis. 

**Figure 1:**
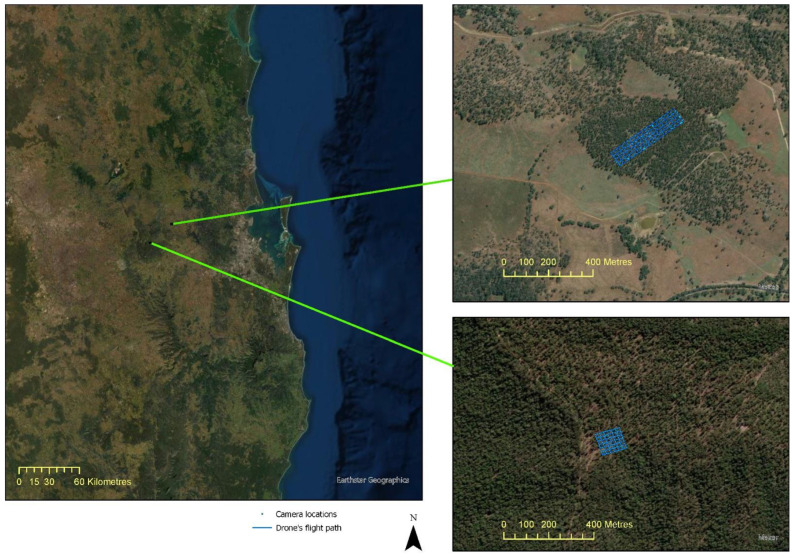
Location of private native forest sites selected for the study and the drone flight path used to collect images. Sites were located near Ravensbourne (site 1) and Esk (site 2). Background true colour images are from different remote-sensing satellites available in ESRI base maps.

Figure 1 shows the location of the private native forest sites selected for the study and the flight path of the drone for collecting the image data. The novel field assessment mapped the individual tree locations using a Trimble GeoXH Geoexplorer, a differential global navigation satellite system (GNSS) with up to 10 cm accuracy. Individual trees were located within existing permanent monitoring plots (generally 40 × 40 m) distributed within different sites selected for the study. For each tree ≥10 cm, diameter at breast height (DBH) and tree height were recorded.

### 3.2. Image Analysis of Drone Images

**Figure 2:**
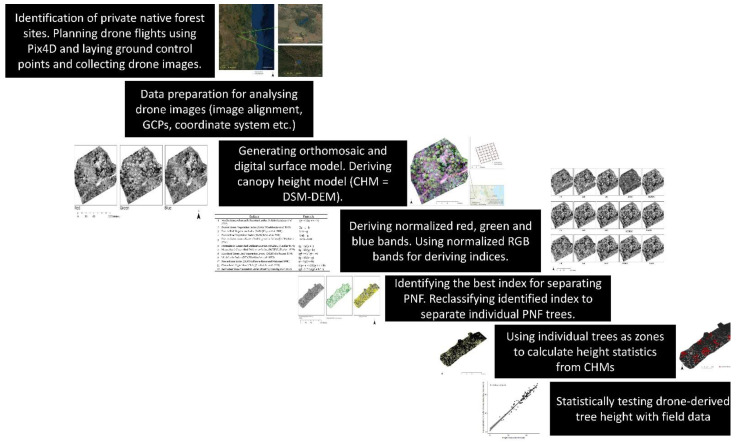
Methodological framework used in this study.

The RGB images collected from the drone were first analysed in Agisoft Metashape software. With multiple overlapping 2d drone images, using structure from motion (SfM), a digital photogrammetry technique, a point cloud and digital surface model (DSM) were generated together with an orthomosaic (Figure 2).

The SfM algorithm is a four-step process where a point of interest is located in each overlapping image. This is automated in software packages such as Metashape. The next step involved finding candidate correspondences with matching descriptors for each interest point. In the third step, a geometric verification of correspondences is performed. This uses the RANSAC algorithm to exclude outliers while matching features. The final step finds a solution for 3d points to minimise reprojection error. This is also referred as bundle adjustment.

An orthomosaic from drone images is a geometrically corrected mosaic that is colour balanced and rectified for distortions due to elevation to create a seamless image of an area. Similarly, the point cloud is a cloud of points with horizontal (x and y) as well as vertical (height) coordinates. Point clouds can easily be interpolated to create a digital raster surface of elevation values.

The RGB orthomosaic was separated into red (R), green (G), and blue (B) spectral bands that were further normalised using the following equations:

(1) $$\mathrm{Normalised}\;\mathrm{red}\;(r)=\frac{R}{R+G+B}$$

(2) $$\mathrm{Normalised}\;\mathrm{green}\;(g)=\frac{G}{R+G+B}$$

(3) $$\mathrm{Normalised}\;\mathrm{blue}\;(b)=\frac{B}{R+G+B}$$

Using the normalised red, green, and blue bands, a number of colour indices were calculated. Table 2 shows a summary of the colour indices, formulas, and references thatwhich were used in the models.

We used ArcGIS Pro 2.8 to calculate all the indices. For this study, we used normalised green-red difference index (NGBDI) to separate vegetation from other features. On visual inspection of all the indices, the normalised green-red difference index (NGBDI) was found best for separating individual trees. Considering this, we used NGBDI to separate trees from other features. Once vegetation was separated, a separate cleaned polygon was generated for vegetation. 

We further used reclassified NGBDI images to generate random locations on the ground surface to generate a digital elevation model (DEM). Using the following equation, we calculated the canopy height model (CHM):

CHM = DSM − DEM(4)

Tree height and tree diameter at breast height (DBH) were measured at 116 locations for 1 of the study areas. The data were collected for site 2 (Figure 1) during field trips in September and November 2020.

To test the accuracy of the tree heights estimated from the drone images, we created a buffer of 1 m around each field data collection location and picked the maximum value from the canopy height model (CHM) within this zone. To test the statistical relationship between drone-derived and the height measured in field, we used a Pearson’s correlation test using the ggpubr library available with R statistics software.

## 4. Results and Discussion

**Figure 3:**
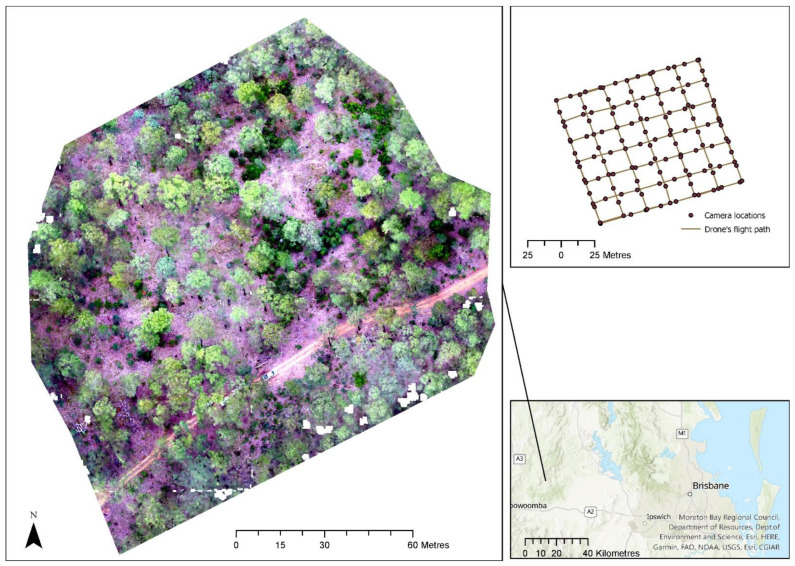
Location of the first site selected for analysing drone data and the orthomosaic of the site.

**Figure 4:**
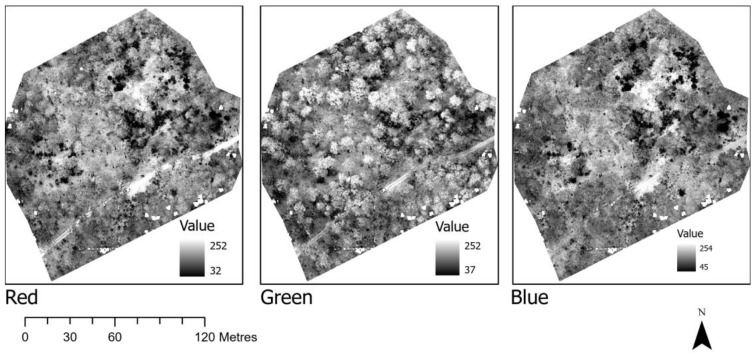
Separation of the orthomosaic into red, green and blue spectrum.

The orthomosaic created for the different sites clearly showed individual trees, especially near the flight path of the drone. Figure 3 shows the location of the first site selected for analysing drone data and the orthomosaic of the site. When the orthomosaic was separated into RGB bands, variation in information content was noticed that was enhanced with the calculation of indices. Figure 4 shows the separation of the orthomosaic into red, green, and blue spectra. The green band showed all the vegetated areas more clearly, while the vegetation was not clearly separated in the blue and green bands (Figure 4).

**Figure 5:**
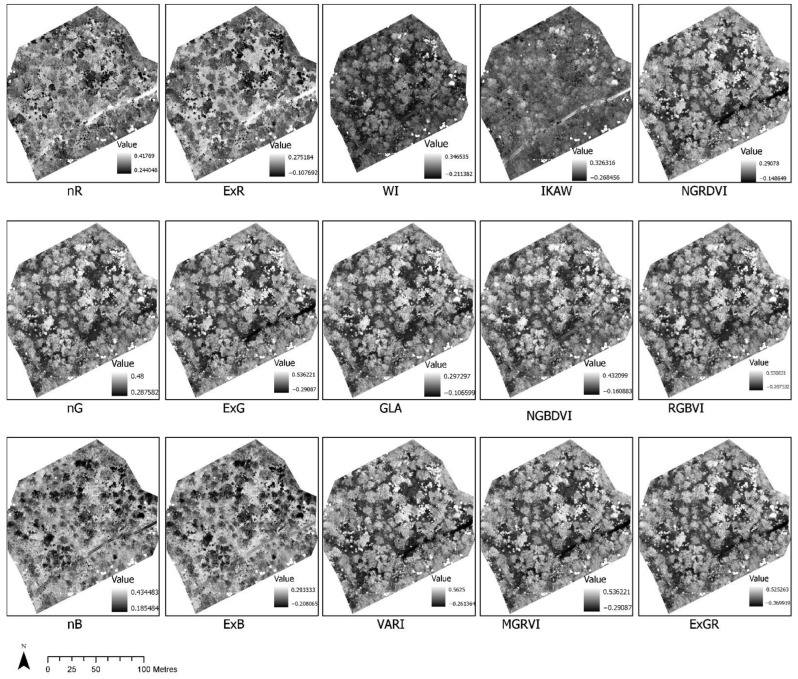
Normalisation of red, green and blue spectrum and calculation of various indices (see Table 2) from normalised bands.

The visual analysis of indices clearly indicated variation in information content that could be further utilised for extracting multiple features such as vegetation types, bare soil, or grass area. For this study, we selected NGBVI to separate vegetation. Figure 5 shows the normalisation of the red, green, and blue spectra and the calculations of various indices from normalised bands.

**Figure 6:**
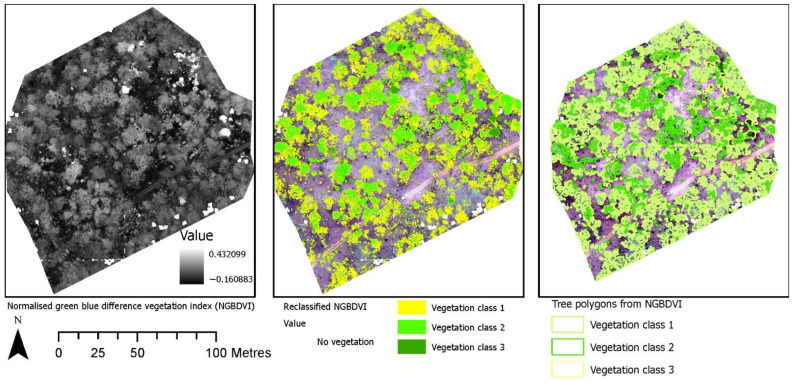
Identification of an index and its reclassification to extract trees and their differentiation into three potential classes, that may represent different tree species.

**Figure 7:**
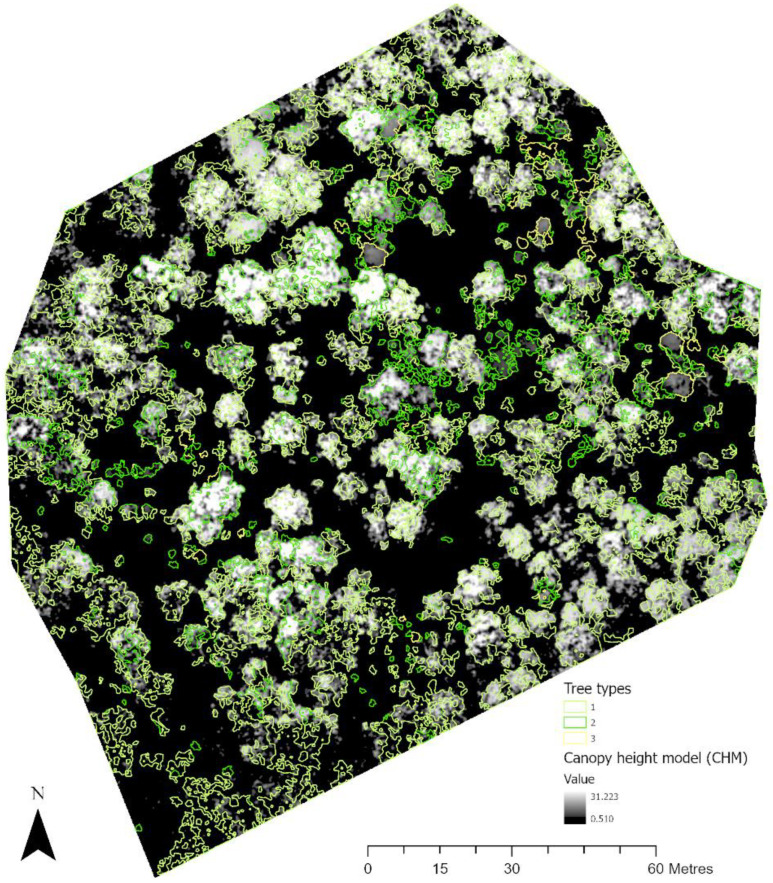
Generation of canopy height model allowing extraction of height information for different trees.

**Figure 8:**
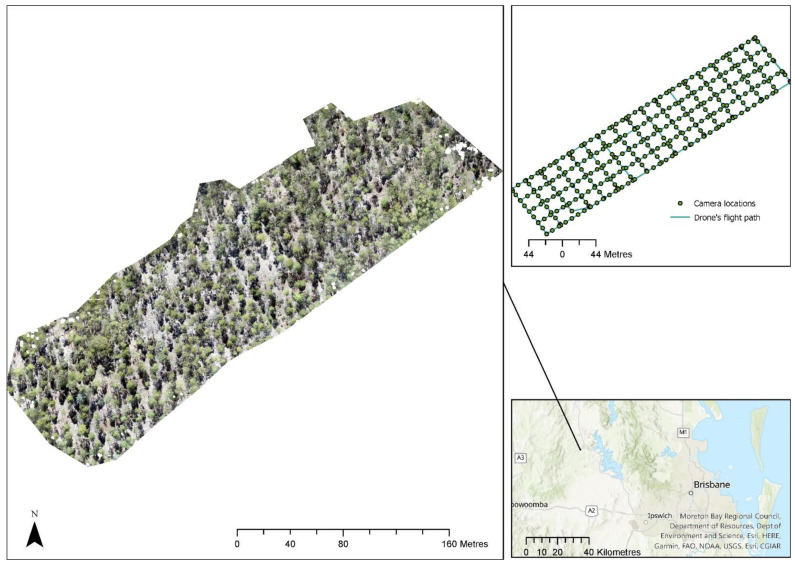
Location of site 2 selected for analysing drone data.

**Figure 9:**
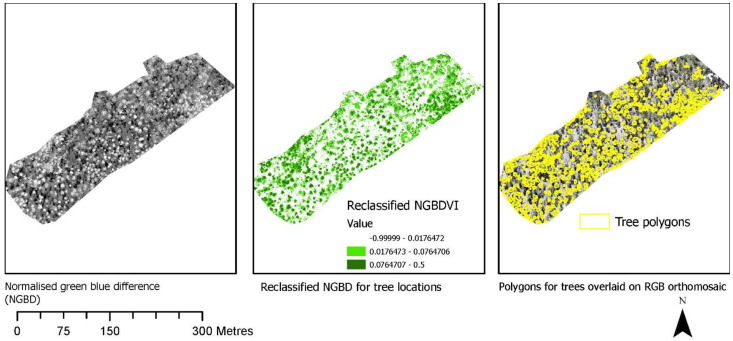
Identification of an index and its reclassification to extract trees and their differentiation into three classes.

**Figure 10:**
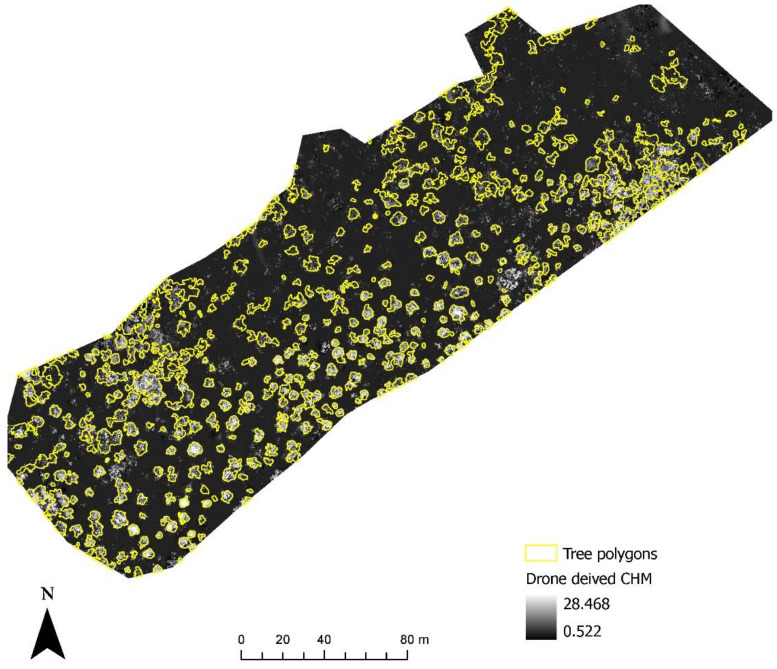
Generating a canopy height model for extraction of height information for different trees.

**Figure 11:**
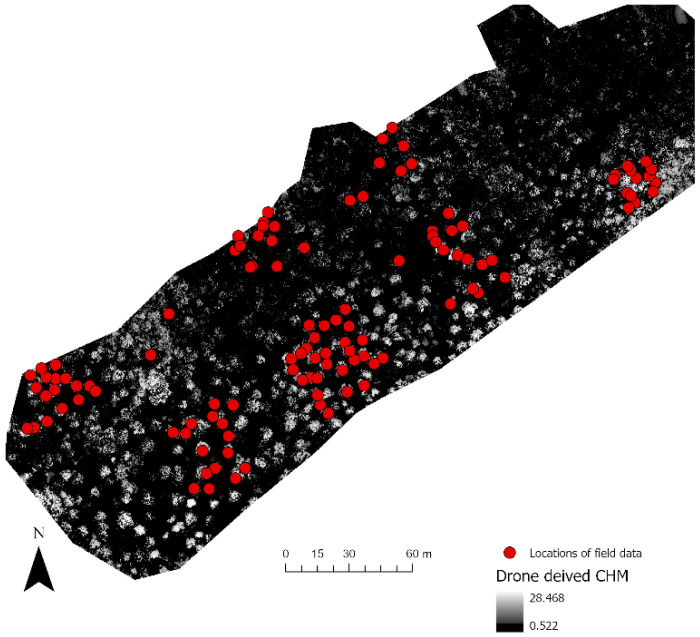
Location of field-measured tree height data points.

**Figure 12:**
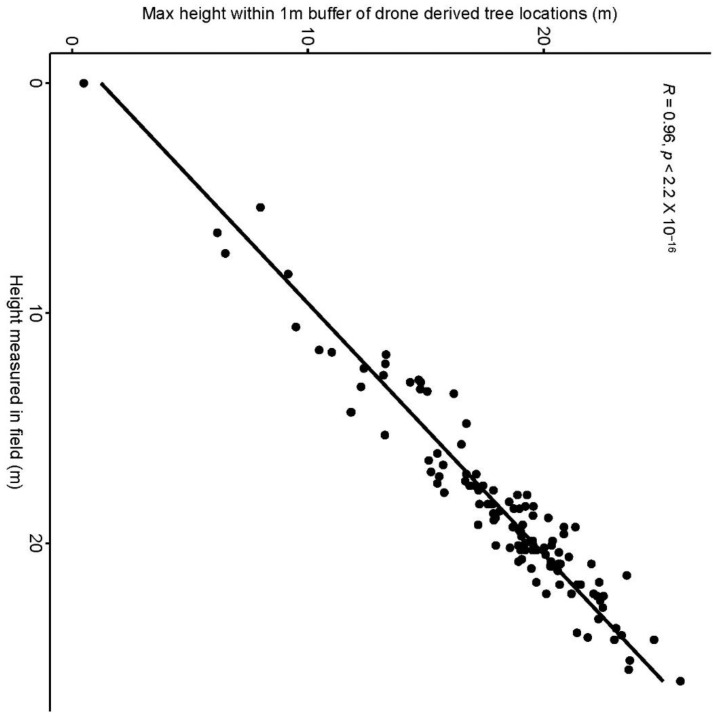
Relationship and statistical test between field-measured and drone-derived tree heights.

Figure 6 shows the identification of an index and its reclassification to extract trees and their differentiation into three classes. For the first site, the reclassification of NGBVI clearly differentiated trees from other features. Variation between three vegetation types based on NGBVI was noted, but this study focuses only on separating trees and estimating their height. The separation of trees enabled the extraction of tree crown areas, which were then converted to simplified polygons with a separate identification. Figure 7 shows the generation of the canopy height model to allow for the extraction of height information for different trees. The polygons created for each tree were further used to perform a zonal operation on CHM to acquire detailed statistics of the height of each tree. Some further results for a second site are shown in Figure 8, Figure 9 and Figure 10. For this site, tree height statistics and crown area were derived, and tree heights were compared with those assessed in the field (Figure 11). There was a strong relationship between field-measured heights and those derived from the CHM (*p* < 0.0001, Figure 12).

## 5. Conclusions

This paper has investigated drone-based and image processing approaches for forest inventory monitoring. The work has shown that vegetated areas and potentially the species contained within can be calculated from indices derived from red, green, and blue bands of orthomosaic images. Furthermore, using CHM data, it was possible to calculate height statistics for vegetated areas. The combination of RGB-derived indices and a canopy height model (CHM) enabled obtaining detailed inventory information for individual trees within PNFs. It was possible to calculate height statistics for individual trees with reasonable accuracy, although this would be more challenging in dense forests with overlapping tree crowns. A few plots were affected by tree shadow, which interfered with other information, although this was minimised to a greater extent with the calculation of indices. This exploratory study suggested that using a combination of different indices and the canopy height model, it will be possible to apply machine and deep learning algorithms to separate vegetation types or species. Future work will investigate this approach and also estimate forest biomass using height information and the area occupied by different trees.

## Figures and Tables

**Table 1:** Some recent works on drone-based approaches for environmental monitoring.

Domain Area	Year	Environmental Application	Techniques Applied	Reference
Land monitoring and identification	2022	Identification of water erosion in mining restored areas	The generation of a digital elevation model (DEM) from drone images and photogrammetric processing	Padro et al., 2022 [22]
	2021	Monitoring of rare plant species	Neural network object detector (YOLO) and darknet neural network framework	Reckling et al., 2021 [23]
	2018	Monitoring of natural/protected reserves from illegal activities	CNN (VGG16, VGG19) and transfer learning to classify four terrain classes: water, deforesting, forest and buildings	Thomazella et al., 2018 [24]
Aquatic/marine monitoring and identification	2021	Detection and quantification of algal in water ecosystems	Image analysis of drone-based multispectral imagery	Toth et al., 2021 [25]
	2020	Drone-based fluorosensor for marine environment monitoring	Analysis of hyperspectral lidar data and fluorescence spectral recordings for vegetation profiling	Duan et al., 2020 [26]
	2016	Mapping of coastal fish nursery grounds and marine habitats	Image segmentation tools (ArcGIS, MultiSpec, eCognition Developer) to classify marine terrain classes: coarse sand, fine sand, leaves, matte, shallow rock, deep rock	Ventura et al., 2016 [27]

**Table 2:** Colour indices, formulas, and references.

	Indices	Formula
1	Visible Atmospherically Resistant Index (VARI) (Gitelson et al. 2002)	(g − r)/(g + r − b)
2	Excess Green Vegetation Index (ExG) (Woebbecke et al. 1995)	2g − r − b
3	Excess Red Vegetation Index (ExR) (Meyer et al. 2008)	1.4r − g
4	Excess Blue Vegetation Index (ExB) (Mao et al. 2003)	1.4b − g
5	Excess Green minus Excess Red Vegetation Index (ExGR) (Neto 2004)	ExG − ExR
6	Normalized Green-Red Difference Index (NGRDI) (Tucker 1979)	(g − r)/(g + r)
7	Normalized Green-Red Difference Index (NGBDI) (Tucker 1979)	(g − b)/(g + b)
8	Modified Green Red Vegetation Index (MGRVI) (Tucker 1979)	(g^2^ − r^2^)/(g^2^ + r^2^)
9	Woebbecke Index (WI) (Woebbecke et al. 1995)	(g − b)/(r − g)
10	Kawashima Index (IKAW) (Kawashima and Nakatani 1998)	(r − b)/(r + b)
11	Green Leaf Algorithm (GLA) (Louhaichi et al. 2001)	(2g − r − b)/(2g + r + b)
12	Red Green Blue Vegetation Index (RGBVI) (Bendig et al. 2015)	(g^2^ − b × r)/(g^2^ + b × r)

## Data Availability

Not applicable.
